# P-1592. Diagnostic Stewardship Cutoffs for Urinalysis Results Prior to Performing a Urine Culture: Analysis of Data from a Healthcare System

**DOI:** 10.1093/ofid/ofae631.1759

**Published:** 2025-01-29

**Authors:** Deborah Kupferwasser, Amy Y Kang, Liz Chen, Holly K Huse, Loren G Miller

**Affiliations:** Division of Infectious Diseases, the Lundquist Institute at Harbor-UCLA Medical Center, Torrance, CA, Torrance, California; Chapman University / Harbor-UCLA Medical Center, Irvine, California; The Lundquist Institute at Harbor-UCLA Medical Center, Torrance, California; Harbor-UCLA Medical Center, Torrance, California; Lundquist Institute at Harbor-UCLA Medical Center, Los Angeles, CA

## Abstract

**Background:**

Urinary Tract Infections (UTIs) are commonly overdiagnosed. Some labs conduct urinalysis (UA) as a preliminary screening before proceeding to urine culture to reduce processing unnecessary samples. However, the optimal UA cutoffs for this diagnostic stewardship intervention are not well defined.

**Figure:**
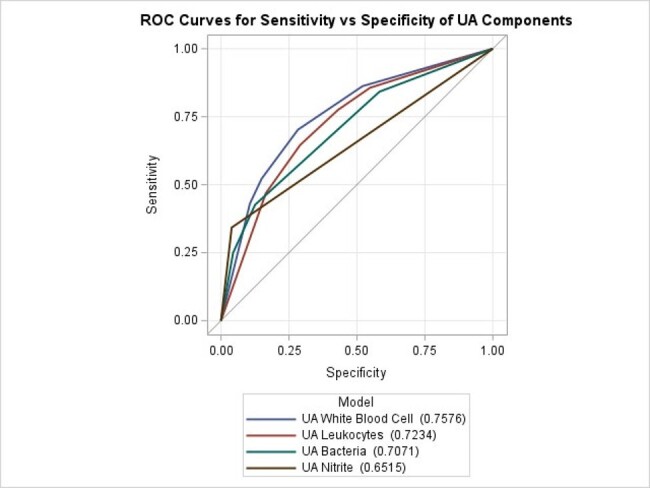

UA squamous epithelial cells were not graphed b/c the value crossed the diagonal at multiple points.

**Methods:**

We performed a retrospective, cross-sectional analysis of urine studies performed from 2/1/21-1/31/23 at the Los Angeles County Department of Health Services (4 medical centers & 20 clinics). We examined patient encounters in which a UA was ordered synchronously with a urine culture. We excluded patients who were pregnant, undergoing a urologic procedure, < 3 months of age, or neutropenic. We examined the diagnostic accuracy, receiver operating characteristic (ROC) curves, and area under the curve (AUC) for pre-screening urinalysis parameters prior to urine culture performance. From the macroscopic test, we examined leukocytes and nitrite. From the microscopic exam, we examined bacteria, white blood cells (WBC), and squamous epithelial cells. We investigated each test’s correlation with a uropathogen isolated from culture.

Sensitivity and specificity of urinalysis WBC cutpoints for identifying uropathogens

**Figure:**
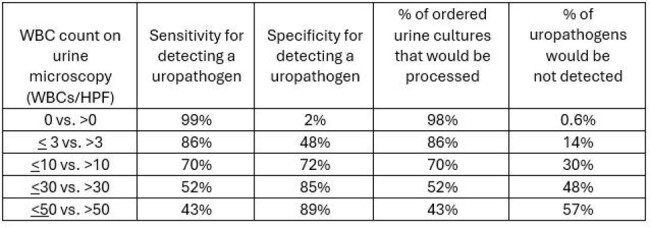

**Results:**

We examined 81,907 paired urinalysis and urine cultures (17,842 inpatient and 64,065 outpatient). Among the urine cultures, organisms isolated included 24,683 (30%) uropathogens, 4,373 (5%) organisms sometimes considered uropathogens, 3,000 (4%) organisms rarely considered a uropathogen, 30,511 (37%) multiple organisms, and 293 (0.7%) a non-uropathogen of clinical significance; 19,047 (23%) cultures were negative. When predicting a urine culture positive for a uropathogen, the UA parameter with the best diagnostic accuracy was WBCs (AUC: 0.758), followed by leukocytes, bacteria, nitrite, and squamous epithelial cells. (AUC: 0.723, 0.707, 0.652, and < 0.500, respectively) (Figure). The sensitivity and specificity of WBC cutoffs are outlined in the Table.

**Conclusion:**

In a large dataset of paired urinalysis and urine cultures, WBC on microscopic urinalysis showed highest diagnostic accuracy at predicting isolation of a uropathogen on urine culture. Stewardship programs should consider this parameter as the screening test for urine culture performance.

**Disclosures:**

**Amy Y. Kang, Pharm.D., BCIDP**, Paratek Pharmaceutical: Grant/Research Support **Loren G. Miller, MD MPH**, Armata: Grant/Research Support|Contrafect: Grant/Research Support|GSK: Grant/Research Support|Merck: Grant/Research Support|Paratek: Grant/Research Support

